# Periodic Mesoporous Organosilica Coated Prussian Blue for MR/PA Dual‐Modal Imaging‐Guided Photothermal‐Chemotherapy of Triple Negative Breast Cancer

**DOI:** 10.1002/advs.201600356

**Published:** 2016-11-21

**Authors:** Wei Tian, Yunyan Su, Ying Tian, Shouju Wang, Xiaodan Su, Ying Liu, Yunlei Zhang, Yuxia Tang, Qianqian Ni, Wenfei Liu, Meng Dang, Chunyan Wang, Junjie Zhang, Zhaogang Teng, Guangming Lu

**Affiliations:** ^1^ Department of Medical ImagingJinling HospitalNanjing Clinical SchoolSouthern Medical University (Guangzhou)Nanjing210002JiangsuP. R. China; ^2^ Department of Medical ImagingJinling HospitalSchool of MedicineNanjing UniversityNanjing210002JiangsuP. R. China; ^3^ Key Laboratory for Organic Electronics & Information Displays and Institute of Advanced MaterialsNanjing University of Posts and TelecommunicationsNanjing210046JiangsuP. R. China; ^4^ State Key Laboratory of Analytical Chemistry for Life ScienceSchool of Chemistry and Chemical EngineeringNanjing UniversityNanjing210093JiangsuP. R. China

**Keywords:** imaging‐guided therapy, nanoplatform, periodic mesoporous organosilica, Prussian blue, triple negative breast cancer

## Abstract

Complete eradication of highly aggressive triple negative breast cancer (TNBC) remains a notable challenge today. In this work, an imaging‐guided photothermal‐chemotherapy strategy for TNBC is developed for the first time based on a periodic mesoporous organosilica (PMO) coated Prussian blue (PB@PMO) nanoplatform. The PB@PMOs have organic‐inorganic hybrid frameworks, uniform diameter (125 nm), high surface area (866 m^2^ g^−1^), large pore size (3.2 nm), excellent photothermal conversion capability, high drug loading capacity (260 µg mg^−1^), and magnetic resonance (MR) and photoacoustic (PA) imaging abilities. The MR and PA properties of the PB@PMOs are helpful for imaging the tumor and showing the accumulation of the nanoplatform in the tumor region. The bioluminescence intensity and tumor volume of the MDA‐MB‐231‐Luc tumor‐bearing mouse model demonstrate that TNBC can be effectively inhibited by the combined photothermal‐chemotherapy than monotherapy strategy. Histopathological analysis further reveals that the combination therapy results in most extensive apoptotic and necrotic cells in the tumor without inducing obvious side effect to major organs.

## 1 Introduction

Breast cancer is one of the most common malignancies among women.^1^ Among them, triple‐negative breast cancer (TNBC) characterized by lacking expression of the estrogen receptor, progesterone receptor, and human epidermal growth factor receptor‐2 is extremely aggressive with a high risk of distant metastasis and early recurrence.^2^ Unlike other breast cancer subtypes, molecular targeted therapy is not suitable for TNBC and they normally receive systemic chemotherapy.^3^ However, many TNBC patient cases show poor response to the therapy and no alternative options are available for those who obtained an initial response or drug resistance.^4^, ^5^, ^6^, ^7^ Therefore, development of new therapeutic strategies against TNBC is urgently needed.

Photothermal‐chemical combination therapy strategy has recently been explored as a potent antitumor approach to treat aggressive tumors.^8^, ^9^ Compared to monotherapy, the combination therapy also exhibits encouraging results for TNBC, such as enhancing therapeutic efficacy, increasing overall survival rates, and lowering drug dose and side effects.^10^, ^11^, ^12^, ^13^, ^14^ For example, Feng et al.^15^ demonstrated that photothermal‐chemotherapy can inhibit the proliferation and lung metastasis of TNBC by using cisplatin–polypeptide wrapped gold nanorods. Nie and co‐workers^16^ reported that TNBC xenografts can almost be ablated with minimal side effects via combination therapy by using an amphiphilic copolymer based nanoplatform. However, to the best of our knowledge, the therapeutic strategies for TNBC on previous studies are lack of imaging guidance. For photothermal‐chemical combination therapy of TNBC, imaging‐guided therapy has several advantages to improve the therapeutic efficacy.^17^, ^18^, ^19^, ^20^, ^21^, ^22^, ^23^, ^24^, ^25^ First, imaging could provide the information of the tumor's shape, size, location, and relationship with surrounding tissues, which is helpful for determining treatment position and scope. Second, optimal treatment time can be chosen when the phototherapeutic agents reached to the targeted lesion. Third, the proceeding of diseases after therapy can be timely monitored. Therefore, it is important to integrate imaging and therapy functionalities into a single nanoplatform to treat TNBC. Besides, previous nanoplatforms for TNBC combination therapy are mainly based on organic block‐polymers or inorganic gold nanoparticles. These pure organic or inorganic nanoplatforms limited from poor stability or low drug loading capacity for tumor therapy. Organic–inorganic hybrid materials possess excellent mechanical stability, easy modification property, and good biobehavior for biomedical applications. However, to the best of our knowledge, organic–inorganic hybrid nanoplatforms have not been constructed for TNBC imaging or treatment.

Prussian blue (PB) has been approved by U.S. Food and Drug Administration for treatment of radioactive exposure in clinical practice.^26^ PB is a prototype of mixed‐valence transition metal hexacyanoferrates with the general formula of Fe^III^_4_[Fe^II^(CN)_6_]_3_·nH2O. Because of the absorbance in the near‐infrared (NIR) window, high photothermal conversion efficiency, T1‐weighted magnetic resonance contrasting ability and approved human safety, PB nanoparticles have been explored for magnetic resonance (MR) imaging, photoacoustic (PA) imaging, and photothermal therapy (PTT).^27^, ^28^, ^29^, ^30^, ^31^, ^32^ However, pure PB nanoparticles cannot deliver drug for cancer chemotherapy because of their small micropores and limited loading capacity. Periodic mesoporous organosilicas (PMOs) synthesized via surfactant‐directed sol–gel process has been used for drug delivery because of their uniform and large mesopore size, high surface area, organic groups incorporated frameworks, and excellent biodegradation and biocompatibility.^33^, ^34^, ^35^, ^36^, ^37^, ^38^, ^39^, ^40^, ^41^, ^42^, ^43^ Based on the unique features, we presumed that an imaging‐guided combination therapy strategy can be developed by intergating the advantages of PMO and PB. To the best of our knowledge, PMO coated PB nanoplatforms have not been reported.

In this work, we first reported a multifunctional nanoparticle by coating periodic mesoporous organosilica on PB (PB@PMO) to perform dual‐modality imaging‐guided photothermal‐chemotherapy of TNBC. The PB@PMO nanoplatforms have organic–inorganic hybrid framework, excellent photothermal conversion capacity, high drug loading capacity, and MR/PA dual‐modal imaging ability. After intravenous injection, MR/PA imaging shows the accumulation of the nanoplatforms in the tumor region and the tumor signals. The bioluminescence intensity, tumor volume, and histopathological analysis demonstrate that the photothermal‐chemotherapy can effectively inhibit growth of TNBC and induce extensive apoptotic and necrotic cells.

## 2 Results and Discussion

**Scheme 1:**
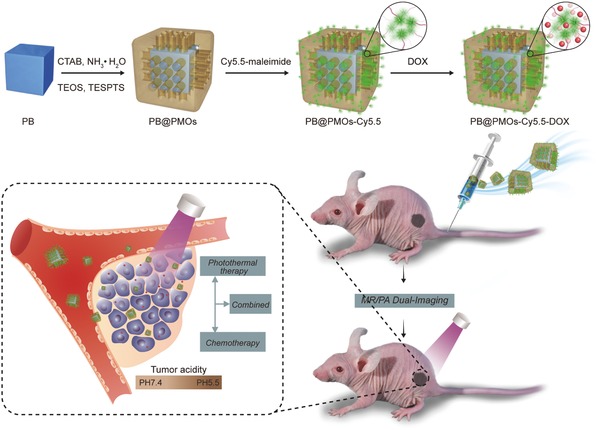
Illustration of the preparation of PB@PMOs‐Cy5.5‐DOX nanoplatforms and the PA/MR dual‐modal imaging‐guided photothermal‐chemotherapy for the TNBC.

The preparation of the PB@PMO nanoparticles and MR/PA imaging‐guided photothermal‐chemotherapy are shown in **Scheme** **1**. First, thioether‐bridged PMOs were coated on the PB nanocubes via a cetyltrimethylammonium bromide (CTAB)‐directed sol–gel process by using tetraethoxysilane (TEOS) and bis(triethoxysily)propane tetrasulfide (TESPTS) as precursors. Then, the thioether group incorporated PB@PMOs were reduced to give thiol groups and then covalently connected with Cy5.5‐maleimide via a click reaction and further loaded with anticancer drug doxorubicin (DOX). After intravenous injection of the obtained PB@PMOs‐Cy5.5‐DOX nanoplatforms, PA and MR dual‐modal imaging and imaging‐guided photothermal‐chemotherapy are performed on TNBC mouse model.

**Figure 1:**
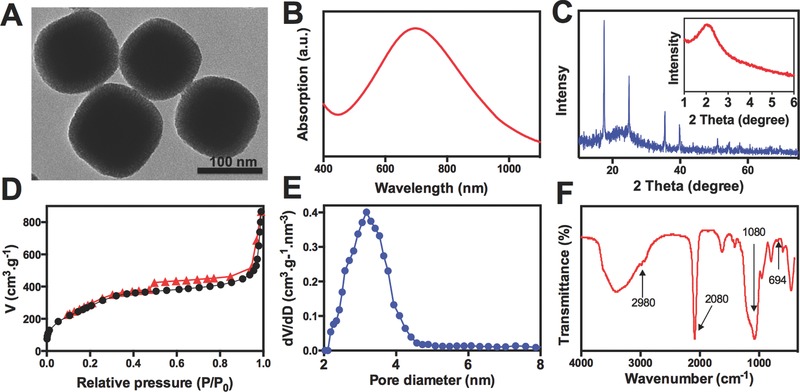
A) TEM image, B) UV–vis absorbance spectrum, C) XRD patterns, D) nitrogen sorption isotherms, E) pore size distribution curve, and F) FT‐IR spectrum of the PB@PMOs.

The transmission electron microscopy (TEM) image showed the PB@PMOs have a well‐defined core–shell structure with a diameter of 125 ± 8 nm and a mesoporous shell of 17 ± 1 nm (**Figure** **1**A). The hydrodynamic diameters of the PB@PMOs in phosphate‐buffered saline (PBS) and cell culture medium are 153 and 158 nm respectively (Figure S1, Supporting Information). The UV–vis absorbance spectrum of the PB@PMOs displayed a strong absorption peak at about 708 nm, which attributed to the encapsulation of PB nanocubes (Figure 1B and Figure S2, Supporting Information). The wide‐angle X‐ray diffraction (XRD) pattern of the PB@PMOs clearly showed the diffraction peaks of PB crystals (Figure 1C and Figure S3, Supporting Information). Simultaneously, the small‐angle XRD pattern of the PB@PMOs showed an obvious peak at 2.04° (Inset in Figure 1C), which is attributed to order mesostructure of the PMO shells. These results demonstrate the successful coating of the PMO shell on the PB nanocube. Nitrogen sorption isotherms of the PB@PMOs showed a type IV curve with a sharp capillary condensation step and a large hysteresis loop in the *p*/*p*_0_ range of 0.47–1.0, indicating that the PMO shell has a typical mesopore architecture with narrow pore size distribution. The surface area and pore volume were calculated to be as high as 866 m^2^ g^−1^ and 0.4 cm^3^ g^−1^, respectively (Figure 1D). The pore size calculated based on the nonlocal density functional theory (NLDFT) revealed the PB@PMO nanoparticles had a uniform mesopore of about 3.2 nm (Figure 1E). The Fourier transform infrared (FT‐IR) spectrum of the PB@PMOs nanoparticles displayed the characteristic Si—O—Si vibration peak at 1080, the C—H absorbance band at 2980 cm^−1^, the C—S band at 694 cm^−1^, and CN group vibration peak at 2080 cm^−1^, furthering confirming the coating of thioether‐bridged PMO shells on PB nanoparticles (Figure 1F and Figure S4, Supporting Information).

**Figure 2:**
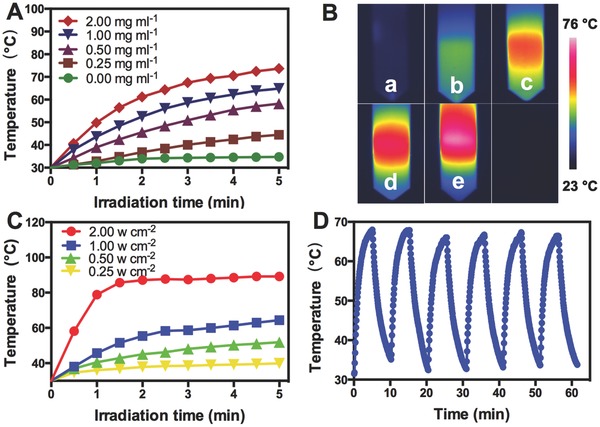
A) Photothermal curves of different concentrations of the PB@PMOs upon an 808 nm laser irradiation at 1.0 W cm^−2^ for 5 min. B) Infrared thermal photographs of the PB@PMOs at different concentrations. (a: 0; b: 0.25 mg mL^−1^; c: 0.50 mg mL^−1^; d: 1.00 mg mL^−1^; e: 2.00 mg mL^−1^). C) Photothermal curves of the PB@PMOs irradiated with different power densities for 5 min at the concentration of 1.0 mg mL^−1^. D) Photostability of the PB@PMOs irradiating at a power density of 1.0 W cm^−2^ for 5 min and cooling down for 10 min for six cycles.

The photothermal property of the PB@PMOs was investigated by exposing the materials to an 808 nm NIR laser at a power density of 1.0 W cm^−2^ for 5 min. It is observed that the temperature increased with the PB@PMOs concentrations under the laser irradiation and rapidly increased to 64.9 °C at the concentration of 1.0 mg mL^−1^ (**Figure** **2**A,B). In contrast, pure water showed only a temperature increase of 4.7 °C. Also, it is showed that the temperature rose from 39.9 °C to 89.2 °C as the power density increased from 0.25 to 2.0 W cm^−2^ at a PB@PMO concentration of 1 mg mL^−1^ (Figure 2C), indicating the temperature increase can also be adjusted by the power density. In addition, photothermal stability of the PB@PMOs was measured. The results showed that the PB@PMOs exhibited excellent photostability after six on–off heating cycles (Figure 2D), demonstrating their promise for PTT.

**Figure 3:**
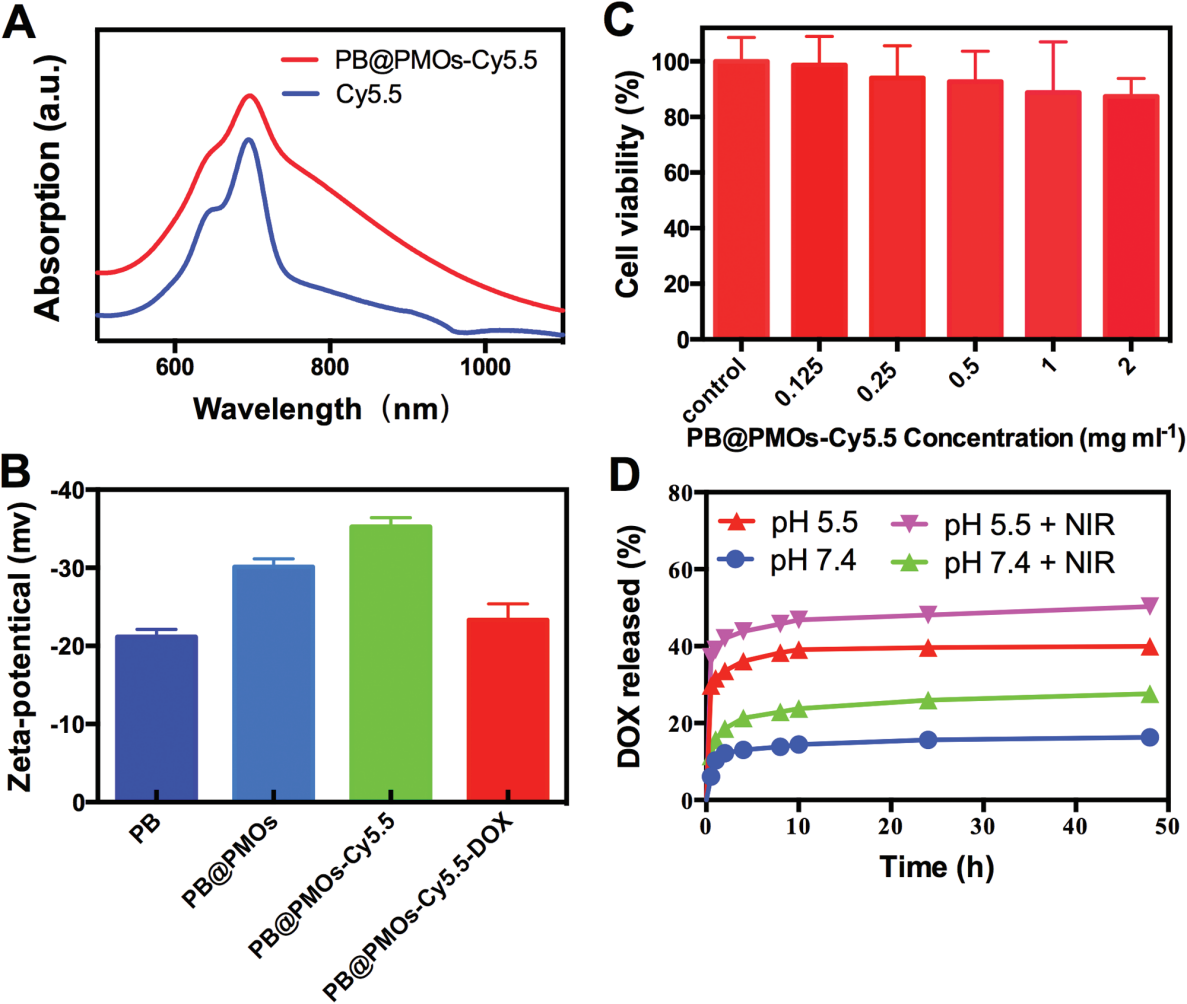
A) UV–vis absorbance spectra of near‐infrared fluorescent dye Cy5.5 and the PB@PMOs‐Cy5.5. B) Zeta potentials of the PB, PB@PMOs, PB@PMOs‐Cy5.5, and PB@PMOs‐Cy5.5‐DOX. C) Relative viability of MDA‐MB‐231 cells incubated with different concentrations of PB@PMOs‐Cy5.5 for 24 h. D) DOX release curves of the PB@PMOs‐Cy5.5‐DOX at pH 7.4 and 5.5 with or without NIR.

To investigate in vitro drug delivery capacity for TNBC cells, the PB@PMOs were modified Cy5.5‐maleimide by reducing the thioether groups to produce thiol groups and then covalently connecting with NIR fluorescent dye Cy5.5‐maleimide via click chemistry. The UV–vis spectrum of the Cy5.5 modified PB@PMOs (denoted as PB@PMOs‐Cy5.5) exhibited the typical absorption peak of fluorescent dye Cy5.5, confirming the successful connecting of Cy5.5 (**Figure** **3**A and Figure S5, Supporting Information). After modification with Cy5.5, the zeta potential of the PB@PMOs turned from −30.1 ± 1.0 mV to −35.3 ± 1.2 mV due to the Cy5.5 is negatively charged (Figure 3B). The cytotoxicity results showed that the relative viabilities of MDA‐MB‐231 cells remained over 87% even after incubation with of the PB@PMOs‐Cy5.5 at a concentration up to 2.0 mg mL^−1^ (Figure 3C), indicating the good compatibility. Then the anticancer drug DOX was loaded into the mesoporous PMO shells to form the PB@PMOs‐Cy5.5‐DOX nanoplatforms. The presence of the DOX absorption peak and the change of the zeta potential confirmed the successful loading of the drug (Figure 3B and Figure S6, Supporting Information). The DOX loaded in the PB@PMOs‐Cy5.5‐DOX was measured to be as high as 260 µg mg^−1^. The drug loading mechanisms are attribution to chemophysical property of PMO. PMO has an open entrance for drugs to enter in, well‐ordered channels for homogeneous distribution of drug molecules. In addition, the surface property is another important factor. The zeta potential of the PB@PMOs is negative charge, while that of the DOX is positive charge. Thus, the DOX can be loaded into PMO via electrostatic interaction. The drug release curves showed that a cumulative DOX release amount of only 16.3% and 27.6 at pH 7.4 without/with NIR in 48 h, while a release amount up to 40.0% and 50.3% at pH 5.5 without/with NIR respectively (Figure 3D). The results indicated that the photo heating and acidic environment could speed up the DOX release rate. Photo heating can speed up the DOX release rate because of accelerating the Brown movement of molecules. As we known, DOX can be loaded in the PMOs via electrostatic interaction in neutral conditions. In acidic environment, the positively charged DOX exchanges with protons, which results in reduction of electrostatic interaction between DOX and PMO. In addition, DOX molecules tend to be more hydrophilic at lower pH values. Therefore, the DOX release profile from the PB@PMO is dependent on pH. It is known that the pH value of tumor microenvironment is lower than normal tissues, thus the pH‐responsive drug release character is benefit for enhancing the drug release at the tumor region and reducing its toxicity to the normal tissues.

**Figure 4:**
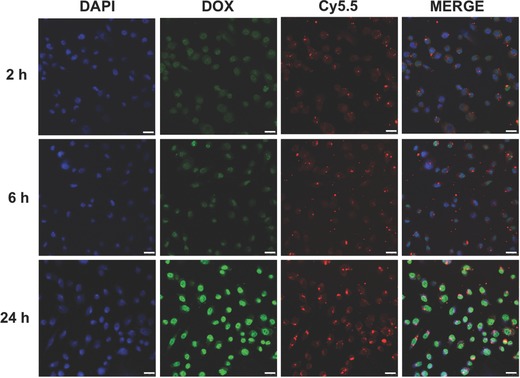
Confocal laser scanning microscopy (CLSM) images of MDA‐MB‐231 cells incubated with 50 µg mL^−1^ of PB@PMOs‐Cy5.5 for 2, 6, and 24 h at 37 °C. The CLSM images of DAPI, DOX, and Cy5.5 were captured under blue (λ_ex_ = 405 nm), red (λ_ex_ = 673 nm), and green (λ_ex_ = 480 nm) fluorescing filters, respectively. Scale bars 25 µm.

We further investigated endocytosis and subcellular localization of the PB@PMOs‐Cy5.5‐DOX and drug release in MDA‐MB‐231 cells. Confocal microscopy analysis showed that both Cy5.5 (red) and DOX (green) signals were presented within cells after incubating MDA‐MB‐231 cells with the nanoplatforms for 2–24 h (**Figure** **4**). The overlay images revealed that fluorescence signal of Cy5.5 was from the cytoplasm of the cells and the DOX fluorescence signal was mainly in the nuclei, suggesting that the nanoplatforms are able to enter into the cells and delivered chemotherapy drug into the nuclei.

**Figure 5:**
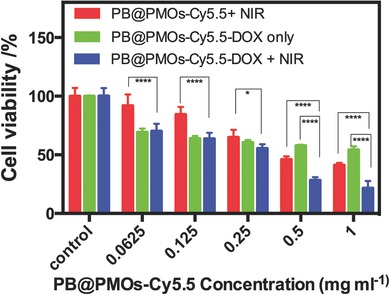
Relative viabilities of MDA‐MB‐231 cells incubated with PB@PMOs‐Cy5.5 and then exposed to a laser irradiation (1.0 W cm^−2^, 5 min) or with PB@PMOs‐Cy5.5‐DOX with or without irradiation.

Next, we studied the in vitro combination photothermal‐chemotherapy of the PB@PMOs‐Cy5.5‐DOX for TNBC cell line MDA‐MB‐231. The results (**Figure** **5**) showed that the cell viability in the PB@PMOs‐Cy5.5 + NIR group decreased with the nanoparticles concentrations. The MDA‐MB‐231 cells incubated with the PB@PMOs‐Cy5.5‐DOX also achieved obvious cytotoxicity and no further cell damage was detected when the particle concentration is up to 0.5 mg mL^−1^. Notably, the PB@PMOs‐Cy5.5‐DOX + NIR induced more lower cell viability than the other groups, which is attributed to combined PTT and chemotherapy. As the concentration increased to 1 mg mL^−1^, the PB@PMOs‐Cy5.5‐DOX + NIR killed 78.6% MDA‐MB‐231 cells.

**Figure 6:**
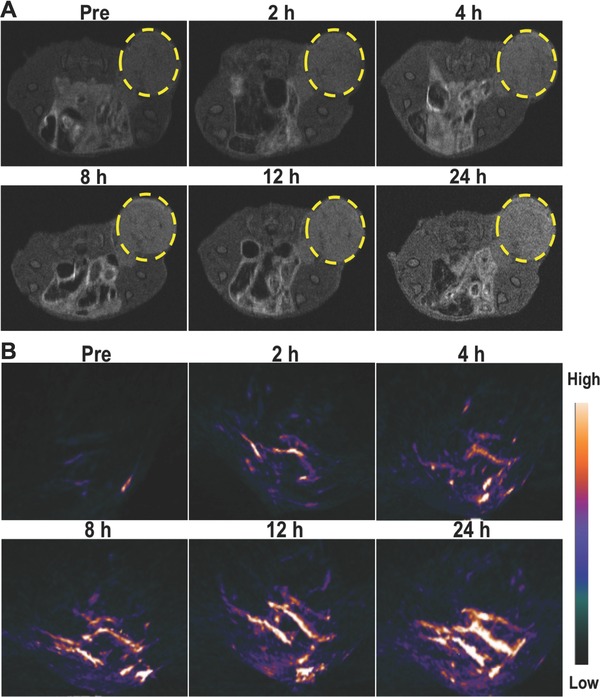
A) T1‐weighted MR and B) PA imaging of MDA‐MB‐231‐Luc tumor‐bearing mice at different time points post injection of PB@PMOs‐Cy5.5.

Because of the encapsulation of the PB, the PB@PMOs has the PA and T1‐weighted MR imaging capability (Figure S7, Supporting Information). Thus we investigated the passive tumor targeting of the PB@PMOs‐Cy5.5 via the dual‐modality imaging. It is observed that the MR signals gradually increased in the tumor region, indicating a time‐dependent tumor accumulation of the PB@PMOs‐Cy5.5 (**Figure** **6**A). In vivo PA imaging also showed that the vascular signals of the tumor gradually enhanced after injecting nanoparticles (Figure 6B), which can be used to guide following therapeutic actions.

**Figure 7:**
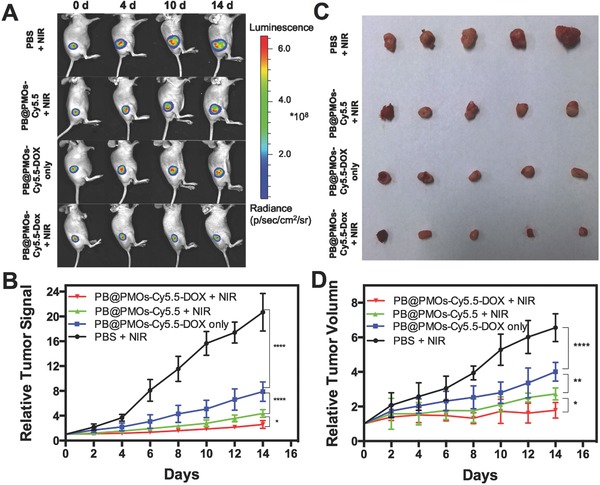
A) Fluorescent photographs, B) relative luciferase intensities, C) representative tumors, and D) growth‐curves of tumor volume of MDA‐MB‐231‐Luc tumor‐bearing mice treated with PBS + NIR, PB@PMOs‐Cy5.5 + NIR, PB@PMOs‐Cy5.5‐DOX only, and PB@PMOs‐Cy5.5‐DOX + NIR.

Further, in vivo combination photothermal‐chemotherapy efficacy of the PB@PMOs‐Cy5.5‐DOX for TNBC was investigated by intravenous injection of the nanoplatform into MDA‐MB‐231‐Luc tumor‐bearing BALB/C female mice. The nanoplatforms can passively accumulate in the tumor site via the enhanced permeability and retention effect. The bioluminescence intensity of the tumor was recorded at every second day to indicate the time course of the tumor growth (**Figure** **7**A,B). On day 14, the relative bioluminescence intensity of the PBS + NIR group increased to 20.7 ± 3.0. In contrast, the bioluminescence intensity of PB@PMOs‐Cy5.5 + NIR and the PB@PMOs‐Cy5.5‐DOX groups increased to 4.4 ± 0.6 and 7.9 ± 1.6, respectively. Particularly, PB@PMOs‐Cy5.5‐DOX + NIR group showed a relative signal of only 2.59 ± 0.62, which is significantly lower than the other groups. Also, the relative tumor volume of the PB@PMOs‐Cy5.5‐DOX + NIR group (1.78 ± 0.45) was less than that of PB@PMOs‐Cy5.5 + NIR (2.72 ± 0.35) (*p* < 0.05), PB@PMOs‐Cy5.5‐DOX only (4.01 ± 0.54) (*p* < 0.0001), and PBS + NIR groups (6.56 ± 0.80) (*p* < 0.0001) (Figure 7C,D). The enhanced efficacy of the PB@PMOs‐Cy5.5‐DOX + NIR group for the TNBC is attributed to the combination therapy, suggesting the potential of the PB@PMOs‐Cy5.5‐DOX nanoplatform for TNBC treatment.

**Figure 8:**
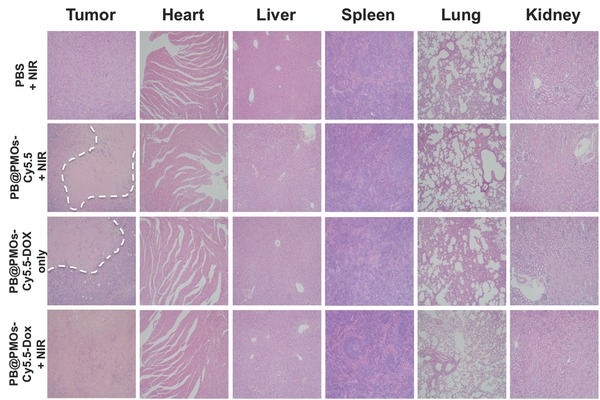
H&E images of tumor and major organs of MDA‐MB‐231‐Luc tumor‐bearing mice after different treatments. The dashed line represents the necrosis areas.

The therapeutic effects and the toxicity of the nanoplatforms were further evaluated by hematoxylin and eosin (H&E) staining. The representative H&E images of the tumor, heart, liver, spleen, lung, and kidney organs of the mice receiving different treatments are shown in **Figure** **8**. Histopathological analysis revealed that the treatment with PB@PMOs‐Cy5.5‐DOX + NIR resulted in the most extensive apoptotic and necrotic cells in the tumor region compared to the other groups. In addition, no noticeable organ damage or inflammatory lesion was observed from H&E stained slices. The results suggested that the combination strategy has excellent cancer therapy efficacy and is safe for major organs.

## 3 Conclusion

In summary, we prepared a multifunctional nanoplatform by coating PMO on PB (PB@PMO) for the first time to perform dual‐modality imaging‐guided photothermal‐chemotherapy of TNBC. The synthesized PB@PMO nanoplatforms have uniform size (125 nm), high surface area (866 m^2^ g^−1^), large pore size (3.2 nm), excellent photothermal conversion capacity, good biocompatibility and high drug loading capacity (260 µg mg^−1^) with a pH‐responsive drug release property. The MR and PA dual‐modal imaging showed that the PB@PMOs gradually accumulated in the TNBC tumor and the signals of tumor and their blood vessels were clearly observed after intravenous injection. The combined photothermal‐chemotherapy significantly inhibited the growth of the MDA‐MB‐231‐Luc tumor compared to solo photothemal or chemical therapy. In addition, histopathological analysis also revealed that the combined photothermal‐chemotherapy resulted in the most extensive apoptotic and necrotic cells in the tumor region. Besides, pathologic examination demonstrated that the combination therapy strategy did not produce obvious toxicity on the main organs. It is believed that the PB@PMO nanoplatforms with dual‐modal PA/MR imaging capacity and photothermal‐chemotherapy effects have great potential for treatment of TNBC.

## 4 Experimental Section

*Materials*: TEOS, CTAB, concentrated ammonia aqueous solution (25 wt%), anhydrous ethanol, dioxane, triphenylphosphine, *N*,*N*‐dimethylformamide (DMF), citric acid, FeCl_3_, K_4_[Fe(CN)_6_]·3H_2_O were bought from Sinopharm Chemical Reagent Co., Ltd. (Shanghai, China). TESPTS was obtained from Sigma‐Aldrich (St. Louis, MO, USA). Cy5.5‐maleimide was purchased from Seebio Biotechnology Co., Ltd. (Shanghai, China). Deionized water (Millipore) with a resistivity of 18 MΩ cm was used in all experiments. Dulbecco's Modified Eagle's Medium, heat‐inactivated fetal bovine serum (FBS), penicillin‐streptomycin solution, and dimethyl sulfoxide were bought from Gibco Laboratories (NY, USA). 3‐(4,5‐Dimethylth‐iazol‐2‐yl)‐2,5‐diphenyltetrazoliumbromide (MTT) was obtained from Nanjing Keygen Biotech. Co., Ltd. (Nanjing, China). TNBC cell lines (MDA‐MB‐231 and MDA‐MB‐231‐Luc) were obtained from American Type Culture Collection.

*Preparation of PB@PMOs Nanoparticles*: PB nanocubes were first synthesized according to Teng et al.^44^ Typically, 40 mL of 1.0 × 10^−3^ m K_4_[Fe(CN)_6_] aqueous solution containing 0.5 mmol citric acid was added dropwisely into 40 mL of 1.0 × 10^−3^ m FeCl_3_ aqueous solution under stirring at 60 °C. Afterward, the products were collected by adding of 80 mL acetone and centrifuged at 15 000 rpm for 15 min. Then, 0.4 mg PB nanocubes were dispersed in a mixed solution containing 0.16 g of CTAB, 25 mL of ethanol, and 80 mL of water. After the solution was heated to 35 °C under vigorous stirring, a mixture of TESPTS (0.01 mL) and TEOS (0.10 mL) was rapidly added. Afterward, 10 µL concentrated ammonia aqueous solution was added. After stirring at 35 °C for 48 h, the products were collected by centrifugation and washed three times with ethanol. Finally, the structure directing agent CTAB was extracted from the as‐synthesized materials three times in a 120 mL solution containing ethanol and concentrated HCl (volume ratio = 500:1) at 60 °C for 3 h. After the sample was washed with ethanol three times and dried in an oven at 40 °C, PB@PMOs were obtained.

*Measurement of Photothermal Effects*: 1 mL aqueous solution of the PB@PMOs at concentrations of 0–2 mg mL^−1^ was held in a quartz cuvette and irradiated with an 808 nm NIR laser at a power density of 1 W cm^−2^ for 5 min. The temperature changes were recorded by using an infrared thermal camera (MAGNITY f15F1, Wuhan VST Light & Technology Co., Ltd., 137 China).

*Modification of Cy5.5 on PB@PMOs*: The S—S bonds in the PMO shells were first reduced to thiol groups according to the method reported in Besson et al.^45^ Typically, the PB@PMOs (62 mg) was dispersed in a mixture of dioxane (1.1 mL), water (0.3 mL), and triphenylphosphine (0.10 g). When the mixture was heated to 40 °C, two drops of concentrated HCl were added under nitrogen. Two hours later, the thiol groups contained PB@PMOs were obtained and dispersed in 2 mL of ethanol. To link NIR dyes, the above obtained suspension (0.5 mL) was added to a mixed solution of Cy5.5‐maleimide (0.1 mg), water (1.0 mL), and DMF (0.1 mL). The mixture was allowed to shake 12 h at room temperature. Finally, the Cy5.5 grafted PB@PMOs were obtained after washing with water.

*Characterization*: TEM images were captured by using a HT7700 microscope (Hitachi, Tokyo, Japan) at 100 kV. The zeta potential and hydrodynamic sizes were measured by using a Brookhaven analyzer (Brookhaven Instruments Co., Holtsville, NY, USA). UV–vis spectra were obtained on a Lambda 35 UV–vis spectrophotometer (PerkinElmer, Inc., Waltham, MA, USA). FT‐IR spectra were recorded on a Nicolet Nexus 870 spectrometer (Nicolet Instruments Inc. Madison, WI, USA). In vivo NIR fluorescence imaging was performed by IVIS Lumina XR system (Xenogen Corporation‐Caliper, Alameda, CA, USA) under the Cy5.5 filter (λ_ex_ = 673 nm, λ_em_ = 707 nm). Nitrogen sorption isotherms were measured by a Micromeritics Tristar 3000 analyzer (Micromeritics Instruments Corporation, Atlanta, GA, USA) at −196 °C. The Brunauer–Emmett–Teller and NLDFT methods were used to calculate the specific surface areas and the pore sizes, respectively. The adsorbed amount at *p*/*p*_0_ = 0.995 was used to estimate the total pore volume.

*Drug Loading and pH‐Responsive Release*: Typically, a mixture of the PB@PMOs‐Cy5.5 (5 mg) and DOX (5 mg) in 10 mL PBS (pH 7.4) was shaken overnight at room temperature. Afterword, DOX loaded PB@PMOs‐Cy5.5 (PB@PMOs‐Cy5.5‐Dox) were obtained by centrifugation and washed five times to remove unloaded DOX. To evaluate the DOX‐loading efficiency, the free DOX content of supernatant solution were calculated using a UV–vis spectrometer at a wavelength of 482 nm. In vitro pH‐responsive DOX releasing was executed in PBS solution at pH 5.5 and pH 7.4. In brief, the above‐prepared PB@PMOs‐Cy5.5‐Dox (2.5 mg) were dispersed in 10 mL of PBS solution at pH 5.5 and pH 7.4 and shook with a speed of 100 rpm at 37 °C. The above mixed solution (0.05 mL) was collected at different time points and centrifuged to remove PB@PMOs‐Cy5.5‐Dox. After that, the supernatant was tested by UV–vis spectroscopy at 482 nm.

*Cell Culture*: The MDA‐MB‐231 cells were cultured at 37 °C under a humidified 5% CO_2_ in Roswell Park Memorial Institute (RPMI) 1640 medium supplemented with 10% FBS, 100 units mL^−1^ penicillin, and 100 mg mL^−1^ streptomycin. The MDA‐MB‐231‐Luc cells were cultured at 37 °C under a humidified 5% CO_2_ in minimum essential medium (MEM) supplemented with 10% FBS, 100 units mL^−1^ penicillin, and 100 mg mL^−1^ streptomycin.

*Cytotoxicity Assay*: The cytotoxicity of the PB@PMOs‐Cy5.5 was analyzed on MDA‐MB‐231 cells. In brief, the cells were seeded in 96‐well plate with a density of 1 × 10^4^ cells per well and incubated with the PB@PMOs‐Cy5.5 at different concentrations (0–2 mg mL^−1^) for 24 h at 37 °C. After that, the standard MTT assay was carried out to determine the cell viability.

*In Vitro Cellular Uptake*: MDA‐MB‐231 cells (1 × 10^5^ cells per well) were planted into a Lab‐Tek Chamber Slide system (Thermo Fisher Scientific, Rochester, NY, USA) in 0.5 mL of RPMI 1640 medium containing 10% FBS under a humidified 5% CO_2_ atmosphere at 37 °C. After incubation overnight, the cells were rinsed with PBS and incubated with new medium (0.5 mL) containing 0.05 mg mL^−1^ PB@PMOs equivalent of PB@PMOs‐Cy5.5‐DOX. After 2, 6, and 24 h, cells were washed several times with PBS and stained by the UltraCruz Mounting Medium (Santa Cruz Biotechnology, TX, USA). The confocal laser scanning microscopy (CLSM) images were performed using an LSM 710 microscope (Carl Zeiss, Germany).

*In Vitro Combined Photothermal‐Chemotherapy*: MDA‐MB‐231 cells were seeded in 96‐well plates at a density of 10^4^ cells per well for 24 h. The cells were incubated with 100 µL culture medium containing PB@PMOs‐Cy5.5 or PB@PMOs‐Cy5.5‐DOX at the PB@PMOs‐Cy5.5 concentrations of 0, 0.0625, 0.125, 0.25, 0.5, and 1.0 mg mL^−1^ for additional 24 h. Afterward, the cells were rinsed with PBS and added with 100 µL of fresh culture medium. The MDA‐MB‐231 cells treated with PB@PMOs‐Cy5.5‐DOX were irradiated with 808 nm laser at a power density of 1.0 W cm^−2^ for 5 min. As the control, the cells were treated with PB@PMOs‐Cy5.5‐DOX without irradiation. Simultaneously, the cell were treated with PB@PMOs‐Cy5.5 and further exposed to an 808 nm laser at a power density of 1.0 W cm^−2^ for 5 min. After that, the cell viability was calculated by MTT method.

*Animal Model*: All animal experiments had been approved by the institutional ethical committee of Jinling Hospital. Female Balb/c mice were purchased from Nanjing Peng Sheng Biological Technology Co. Ltd. MDA‐MB‐231‐Luc cells (5 × 10^6^) suspended in 0.1 mL PBS were subcutaneously injected into the back of each mouse. When the tumor volume reached about 100–150 mm^3^, animal experiments were carried out subsequently.

*In Vivo MR/PA Imaging*: When the model of tumor‐bearing mice was finished, 0.1 mL PB@PMOs‐Cy5.5 with 10 mg mL^−1^ PB@PMO equivalent concentration was injected into each mice via tail vein. MR imaging was conducted by using a 7.0‐T Brucker PharmaScan Micro‐MRI instrument. PA imaging was performed with a PA computerized tomography scanner (Endra Nexus 128, Ann Arbor, MI). The imaging signals at different time points were collected and signal intensity was calculated using software named ImageJ for each mouse.

*In Vivo Combination Therapy*: The MDA‐MB‐231‐Luc tumor‐bearing BALB/C female mice were randomly segregated into four groups (*n* = 5 per group), minimizing the weight and tumor size differences of each group, as follows: group 1: PBS + NIR; group 2: PB@PMOs‐Cy5.5 + NIR; group 3: PB@PMOs‐Cy5.5‐DOX only; group 4: PB@PMOs‐Cy5.5‐DOX + NIR. First, 0.1 mL of PB@PMOs‐Cy5.5 and PB@PMOs‐Cy5.5‐DOX with the PB@PMOs concentration of 10 mg mL^−1^ or PBS alone was injected via tail vein. After 24 h, photothermal therapy was immediately performed on group 1, 2, and 4 by irradiating the tumor regions with an 808 nm laser at a power density of 2.0 W cm^−2^ for 5 min. All animals were imaged by injecting D‐luciferin substrate every two d starting from day 0 until the end of the experiment. Tumor sizes were measured for the maximum width (*X*) and length (*Y*) every 2 d and the tumor volumes (*V*) were calculated using the formula: *V* = (*X* ^2^ *Y*)/2. Changes of tumor volume were determined for each mice by normalizing the tumor volume at day *T* to the respective tumor volume at day 0. At the end of experiment, the tumor, heart, liver, spleen, lung, and kidney tissues of mice from each group were collected and cryosectioned at 7 mm thickness onto slides and stained with H&E according to the manufacturer's instructions.

*Statistical Analysis*: Statistical analysis was performed by two‐sided Student's *t*‐test for two groups, and two‐way analysis of variance for multiple groups using GraphPad Prism 6 (GraphPad Software Inc., CA, USA). Probabilities as *p* < 0.05 (*), *p* < 0.01 (**), *p* < 0.001 (***), *p* < 0.0001 (****), and no significance (n.s.) were marked in each figure.

## Supporting information

As a service to our authors and readers, this journal provides supporting information supplied by the authors. Such materials are peer reviewed and may be re‐organized for online delivery, but are not copy‐edited or typeset. Technical support issues arising from supporting information (other than missing files) should be addressed to the authors.

Supplementary
