# Supplementary material for: Periodic Mesoporous Organosilica Coated Prussian Blue for MR/PA Dual‐Modal Imaging‐Guided Photothermal‐Chemotherapy of Triple Negative Breast Cancer
Source: Adv Sci (Weinh). 2016 Nov 21;4(3):1600356. doi: 10.1002/advs.201600356 (PMC5357980; doi:10.1002/advs.201600356)
Supplement: Supplementary file 1 — Supplementary [file ADVS-4-na-s001.pdf]

Copyright WILEY-VCH Verlag GmbH & Co. KGaA, 69469 Weinheim, Germany, 2016.

Supporting Information

for *Adv. Sci.,* DOI: 10.1002/advs.201600356

Periodic Mesoporous Organosilica Coated Prussian Blue for MR/PA Dual-Modal Imaging-Guided Photothermal-Chemotherapy of Triple Negative Breast Cancer

Wei Tian, Yunyan Su, Ying Tian, Shouju Wang, Xiaodan Su, Ying Liu, Yunlei Zhang, Yuxia Tang, Qianqian Ni, Wenfei Liu, Meng Dang, Chunyan Wang, Junjie Zhang, Zhaogang Teng, and Guangming Lu**

Copyright WILEY-VCH Verlag GmbH & Co. KGaA, 69469 Weinheim, Germany, 2013.

Supporting Information

Periodic Mesoporous Organosilica Coated Prussian Blue Nanocube for MR/PA Dual-modal Imaging-Guided Photothermal-Chemotherapy of Triple Negative Breast Cancer

Wei Tian, Yunyan Su, Ying Tian, Shouju Wang, Xiaodan Su, Ying Liu, Yunlei Zhang, Yuxia Tang, Qianqian Ni, Wenfei Liu, Meng Dang, Chunyan Wang, Junjie Zhang, Zhaogang Teng and Guangming Lu**

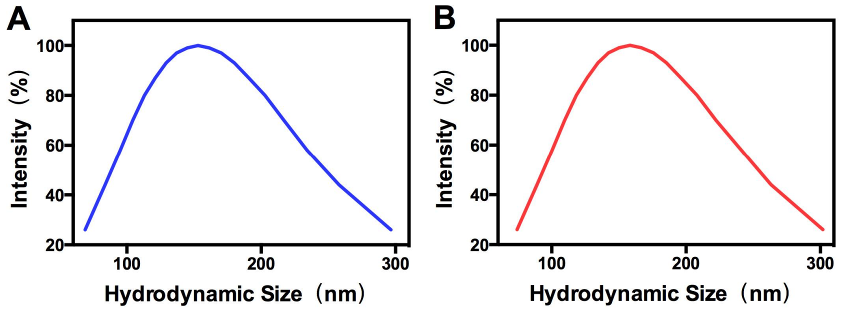

Figure S1. The hydrodynamic diameter of the PB@PMO in the PBS (A) and cell culture medium (B).

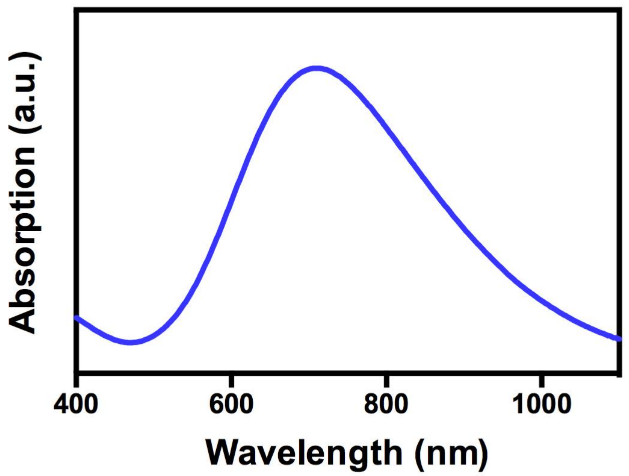

Figure S2. UV-vis absorbance spectra of PB nanocubes.

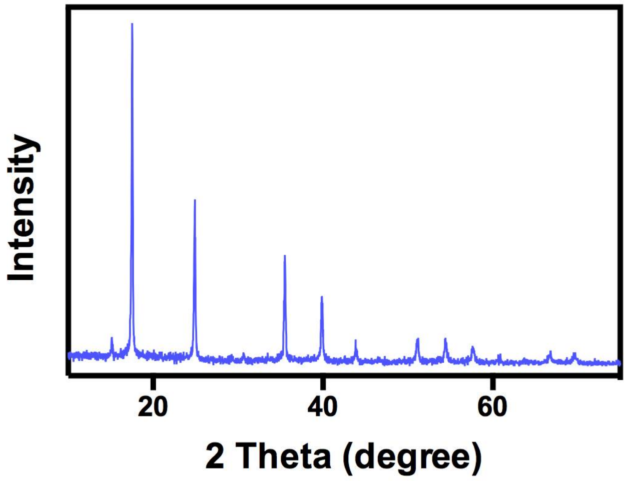

Figure S3. XRD pattern of PB nanocubes.

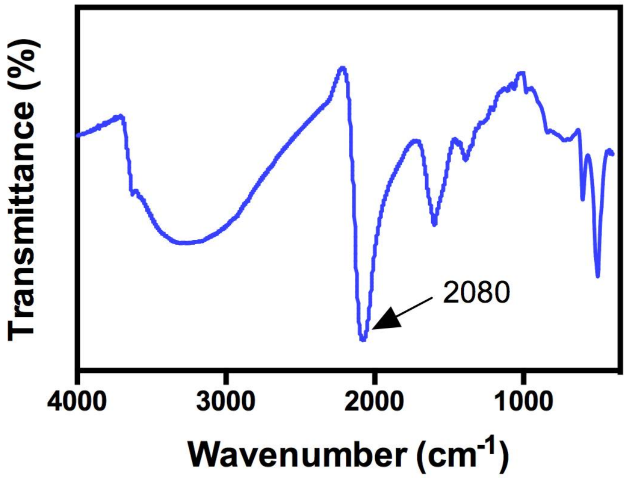

Figure S4. FT-IR spectra of PB nanocubes.

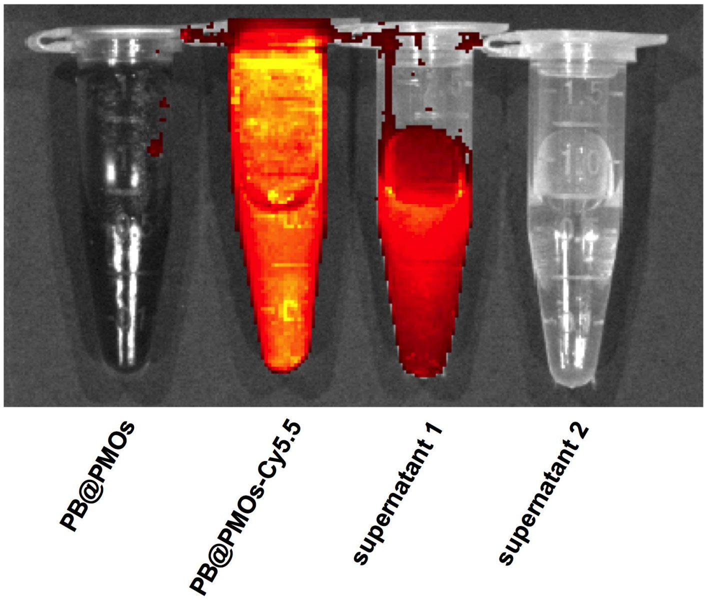

Figure S5. The fluorscence imaging of PB@PMOs, PB@PMOs-Cy5.5, the supernatant of the reaction solution of PB@PMOs and Cy5.5-maleimide (supernatant 1) and the supernatant of the obtained PB@PMOs-Cy5.5 after washing with water for one (supernatant 2). The results confirmed the successful connecting of Cy5.5 on the PB@PMOs.

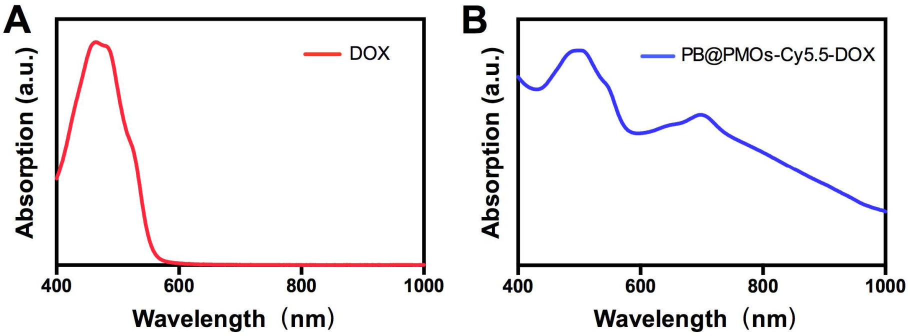

Figure S6. UV-vis absorbance spectra of (A) DOX and (B) PB@PMOs-Cy5.5-DOX.

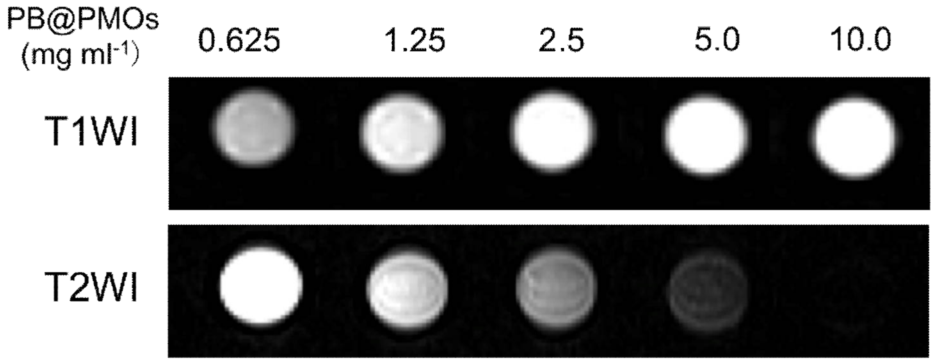

Figure S7. T1 and T2 weighted MR images of the PB@PMOs at different concentrations.
